# Editorial: More on Phytomelatonin: Metabolism and Physiological Roles

**DOI:** 10.3389/fpls.2022.890885

**Published:** 2022-04-25

**Authors:** Frank Bedon, Haitao Shi, Lauren A. E. Erland, Marcello Iriti, Jie Zhou

**Affiliations:** ^1^Department of Animal, Plant and Soil Sciences, School of Agriculture, Biomedicine and Environment, La Trobe University, Bundoora, VIC, Australia; ^2^Hainan Key Laboratory for Sustainable Utilization of Tropical Bioresources, College of Tropical Crops, Hainan University, Haikou, China; ^3^Chemistry, University of British Columbia, Kelowna, BC, Canada; ^4^Department of Agricultural and Environmental Sciences, Università degli Studi di Milano, Milan, Italy; ^5^Department of Biomedical Surgical and Dental Sciences, Università degli Studi di Milano, Milan, Italy; ^6^Department of Horticulture, Laboratory, Institute Zhejiang University, Hangzhou, China

**Keywords:** melatonin, plant, metabolism, physiology, stress responses

Melatonin (N-acetyl-5-methoxytryptamine) is a tryptophan-derived indole amine molecule present in animals, plants, and microbes. It was first identified in animals in 1958 where it mediates the regulation of circadian and seasonal rhythms. Melatonin and its derivatives protect animal cells by scavenging many types of reactive oxygen and nitrogen species (ROS/RNS) and up-regulating the expression of antioxidative enzymes. It also can act as an anti-inflammatory and immunomodulator, with the potential to reduce the severity of disease-associated symptoms.

Discovered in plants a quarter of a century ago, phytomelatonin has been reported from many mono- and dicotyledonous species. Research investigating the effects of exogenous melatonin application and gene expression of melatonin-related genes indicate that the endogenous levels of phytomelatonin can modulate many aspects of plant growth and can have a protective role by reducing the negative effects of biotic and abiotic stresses.

This Research Topic focuses on phytomelatonin metabolism and physiological roles in planta and is a follow up on “Melatonin in plants.” We aim to ask how melatonin alone or in association with other melatonin-derived compounds can modulate plant physiology and what other role(s) they may have. This Research Topic contains one review and 12 original research studies. The articles describe findings that further elucidate the role of phytomelatonin in biotic and abiotic stress tolerance, including some of the molecular actors and mechanism associated to phytomelatonin biosynthesis.

The review by Murch and Erland employed a systematic approach using the PRISMA protocol to highlight the current state of research published in plant physiology, growth, and metabolism since its discovery in plants in 1995. An active and exciting area of phytomelatonin research has been in enhancing understanding pathway dynamics of melatonin biosynthesis in plants with the recent discoveries of alternate pathways and biologically important melatonin isomers. Zheng et al. and Zhou et al. have characterized and identified acetylserotonin-O-methlytransferase (ASMT) and serotonin-N-acetyltransferase (SNAT) in mulberry (*Morus alba* L.) and St. John's wort (*Hypericum perforatum* L.) respectively. In mulberry, ASMT was found to be able to catalyze production of melatonin as well as two isomers described as melatonin isoform (MI)-1 and MI-2, with differential distribution of these molecules across *Morus* species and tissues surveyed. In St. John's wort an early model for phytomleatonin research two leaf specific HpSNAT are described which confer salt and drought tolerance in *Arabidopsis* transgenic plants overexpressing HpSNAT.

Tolerance to abiotic and biotic stresses are investigated in this Research Topic and are key roles of melatonin in plants. Zhu et al. reported an enhanced resistance to *Botrytis cinerea* infection in *Arabidopsis* with overexpression of SNAT and ASMT, the final two steps in conversion of serotonin to melatonin, while susceptibility increased when silenced. This study is of particular interest as it slows for differentiation between serotonin and melatonin mediated effects. Interestingly jasmonic acid (JA) content was also increased with *SNAT* and *ASMT* expression levels in transgenics following fungal infection highlighting the role of phytomelatonin in the activation of JA signaling pathway.

Abiotic stress responses are examined for several species. In rice, Li et al. found that melatonin treatment improved seed germination at low temperature through activation of gibberellin biosynthesis, by maintaining the redox homeostasis, and endogenous melatonin biosynthesis, but prevented the buildup of abscisic acid (ABA) and hydrogen peroxide. Moreover, the authors demonstrated that melatonin acts synergistically with an ABI5-mediated signal involving the regulation of the rice *CATALASE2*. In tomato, Ding et al. found that, while the cold stress increased JA accumulation, the biosynthesis of melatonin was also increased *via* MYC2-activated SlSNAT and SlASMT therefore potentiating cold tolerance. Yang N. et al. reported that selenite treatment at low concentration improved the cold tolerance of cucumber seedlings and increased endogenous melatonin content through up-regulation of key melatonin biosynthetic genes. The research by Xing et al. on heat-resistance mediated by melatonin in chrysanthemum seedlings employed physiological and transcript analyses to reveal the underlying associated gene regulatory networks such as osmotic regulation, redox homeostasis, hormone signal transduction and the chlorophyll, flavonoid, and carotenoid metabolic pathways. In tomato, Jahan et al. found that the attenuation of heat stress induced senescence by melatonin treatment involved enhanced gibberellic acid (GA) and endogenous melatonin content but reduced abscisic acid, also evidenced using gene transcript levels. Furthermore, chemical inhibition of GA and ABA synthesis failed to produce melatonin induced heat tolerance. In tobacco, Chen, Jia et al. investigated the regulatory mechanisms by which melatonin treatment improve dehydration-induced leaf senescence in seedlings and found that foliar spraying decreased endogenous ABA content and oxidative damage. Moreover, metabolite profiling and gene expression analysis showed that melatonin induces carotenoids, inhibits chlorophyll breakdown, modulates phytohormonal biosynthesis and signaling as well as the transcriptional network underlying leaf senescence. Yang S-J. et al. reported that the high light stress response in Arabidopsis leaf was improved with exogenous melatonin application by enhancing the endogenous melatonin content, protecting the photosynthetic pigments and integrity of photosynthesis apparatus, and inhibiting ROS accumulation. In wheat, Zhang et al. compared three winter wheat varieties with contrasted responses for salt tolerance and reported the promotive effect of exogenous melatonin on germination trait under salt stress. Improved wheat germination by melatonin was found to be related to the increase in root vigor, maintenance of ion balance, decrease in H_2_O_2_ content, regulation of soluble protein and sugar synthesis, and changes in amino acid levels. Chen, Cao et al. found that application of melatonin on alfalfa plants submitted to high-nitrate stress improved growth and development by regulating the excess nitrogen and calcium intake, promoting nitrogen metabolism with enhanced enzyme activities, reducing ATP-utilizing system and increasing ATP regeneration.

Together this Research Topic provides readers with overview of the key roles melatonin plays in plants. It highlights innovative and growing areas in the discipline and opens new pathways of discovery that we trust readers will find inspiring.

## Author Contributions

FB wrote and revised the manuscript. HS, LE, MI, and JZ provided suggestions and revised the manuscript. All authors approved the manuscript and the version to be published.

## Funding

This research was supported by the National Key Research and Development Program of China (2019YFD1000300) and the Starry Night Science Fund of Zhejiang University Shanghai Institute for Advanced Study (SN-ZJU-SIAS-0011).

## Conflict of Interest

The authors declare that the research was conducted in the absence of any commercial or financial relationships that could be construed as a potential conflict of interest.

## Publisher's Note

All claims expressed in this article are solely those of the authors and do not necessarily represent those of their affiliated organizations, or those of the publisher, the editors and the reviewers. Any product that may be evaluated in this article, or claim that may be made by its manufacturer, is not guaranteed or endorsed by the publisher.

